# Is 99mTc-diphosphonate uptake the earliest sign of cardiac amyloidosis development in asymptomatic Glu89Gln transthyretin gene mutation carriers?

**DOI:** 10.1186/1750-1172-10-S1-P62

**Published:** 2015-11-02

**Authors:** Fabio Minutoli, Anna Mazzeo, Gianluca Di Bella, Giuseppe Vita, Sergio Baldari

**Affiliations:** 1 University of Messina, Department of Biomedical Sciences and of Morphologic and Functional Images, 98125, Messina, Italy; 2 University of Messina, Department of Neurosciences, 98125, Messina, Italy; 3 University of Messina, Clinical and Experimental Medicine Department, 98125, Messina, Italy

## Background

Presenting symptoms in patients with Glu89Gln transthyretin (TTR) gene mutation are related to peripheral and autonomic nervous system damage; nevertheless, Glu89Gln TTR gene mutation is responsible for early and severe cardiac involvement (which significantly worsens the prognosis). Early diagnosis of cardiac involvement in subjects with TTR gene mutation can significantly affect patient therapy.

We compared 99mTc-3, 3-diphosphono-1, 2-propanodicarboxylic acid (DPD) imaging with electrocardiography (ECG), echocardiography, biomarkers dosage (N-terminal pro–B-type natriuretic peptide (NT-proBNP) and troponin-I) and magnetic resonance (MR) imaging with late gadolinium enhancement (LGE) in order to determine the most sensitive technique in early detection of cardiac amyloid deposition in subjects with Glu89Gln TTR gene mutation.

## Methods

Seven asymptomatic subjects (3M and 4F; mean age, 42 years) with Glu89Gln TTR gene mutation and normal interventricular septum (IVS) thickness and NT-proBNP level underwent three 99mTc-DPD scans (at baseline and two and four years later) and were followed-up for 5-8 years by clinical examination, ECG, echocardiography and cardiac biomarkers dosage. Baseline MR imaging with LGE was also available.

Scintigraphic images were analyzed visually (grade 0, no abnormal localization of the radiotracer; grade 1, myocardial radiotracer uptake lower than bone uptake; and grade 2, myocardial radiotracer uptake higher than bone uptake) and semiquantitatively.

## Results

Three patients showed no myocardial accumulation in all 99mTc-DPD scans; increased IVS thickness occurring four years after the last 99mTc-DPD scan was the only abnormal finding in these patients. In two patients, 99mTc-DPD scan revealed grade 2 radiotracer uptake; baseline MR imaging showed focal LGE in both patients. In these patients, mean left ventricle (LV) wall thickness >12 mm occurred within 3 years; NT-proBNP reached the current diagnostic level for cardiac amyloidosis in only one patient, six years after the positive scan. Two patients had negative baseline 99mTc-DPD scan and cardiac uptake in the following scans. Increased mean LV wall thickness was found three years after positive scintigraphy; NT-proBNP increased later in one patient. ECG abnormalities appeared some years after a positive 99mTc-DPD scan had occurred.

## Conclusion

Cardiac uptake of 99mTc-DPD precede clinical, instrumental and laboratory signs of amyloidosis; it may represent the earliest sign of cardiac amyloidosis development in subjects with Glu89Gln TTR gene mutation preceding of some years fulfillment of current diagnostic criteria for cardiac amyloidosis.

